# Recurrent pneumonia among Japanese adults: disease burden and risk factors

**DOI:** 10.1186/s12890-016-0359-1

**Published:** 2017-01-11

**Authors:** Tomoko Ishifuji, Eiichiro Sando, Norihiro Kaneko, Motoi Suzuki, Paul E. Kilgore, Koya Ariyoshi, Konosuke Morimoto, Naoto Hosokawa, Makito Yaegashi, Masahiro Aoshima, on behalf of the Adult Pneumonia Study Group - Japan (APSG-J), Masahiko Abe, Takao Wakabayashi, Masahiro Aoshima, Naoto Hosokawa, Norihiro Kaneko, Naoko Katsurada, Kei Nakashima, Yoshihito Otsuka, Eiichiro Sando, Kaori Shibui, Daisuke Suzuki, Kenzo Tanaka, Kentaro Tochitani, Makito Yaegashi, Masayuki Chikamori, Naohisa Hamashige, Masayuki Ishida, Hiroshi Nakaoka, Norichika Aso, Hiroyuki Ito, Kei Matsuki, Yoshiko Tsuchihashi, Koya Ariyoshi, Bhim G. Dhoubhadel, Akitsugu Furumoto, Sugihiro Hamaguchi, Tomoko Ishifuji, Shungo Katoh, Satoshi Kakiuchi, Emi Kitashoji, Takaharu Shimazaki, Motoi Suzuki, Masahiro Takaki, Konosuke Morimoto, Kiwao Watanabe, Lay-Myint Yoshida

**Affiliations:** 1 Department of Clinical Medicine, Institute of Tropical Medicine, Nagasaki University, Sakamoto 1-12-4, Nagasaki, 852–8523, Japan; 2 Department of Clinical Tropical Medicine, Graduate School of Biomedical Sciences, Nagasaki University, Nagasaki, Japan; 3 Department of General Internal Medicine, Kameda Medical Center, Kamogawa, Chiba Japan; 4 Department of Pulmonology, Kameda Medical Center, Kamogawa, Chiba Japan; 5 Department of Family Medicine and Public Health Sciences, School of Medicine, Wayne State University, Michigan, USA; 6 Department of Infectious Diseases, Kameda Medical Center, Kamogawa, Japan

**Keywords:** Recurrent pneumonia, Community-acquired pneumonia, Elderly, Epidemiology

## Abstract

**Background:**

In Japan and other societies with rapidly aging populations, recurrent pneumonia (RP) is a major clinical problem yet only limited information exists regarding the burden of this disease.

**Methods:**

A prospective study of adult pneumonia was conducted to investigate the incidence of RP and potential risk factors. From February 1, 2012 to January 31, 2013, patients aged ≥ 15 years who were diagnosed with pneumonia were prospectively enrolled in a representative community hospital located in central Japan. Patients were followed for one-year to evaluate the recurrence of pneumonia and characteristics associated with RP. Cox proportional hazards models were constructed to compute adjusted hazard ratios (aHR) and ascertain risk factors significantly associated with RP.

**Results:**

In total, 841 patients with a median age of 73 years (range 15–101 years) were enrolled totaling 1,048 person-years of observation with a median follow-up time of 475 days. A total of 137 patients had at least one recurrent episode with an incidence rate of 13.1 per 100 person-years (95% confidence interval: 11.1–15.5). In multivariate analysis, a past history of pneumonia (aHR 1.95, 95% CI: 1.35–2.8), chronic pulmonary disease (aHR 1.86, 1.24–2.78) and inhaled corticosteroid usage (aHR 1.78, 1.12–2.84) and hypnotic/sedative medication usage (aHR 2.06, 1.28–3.31) were identified as independent risk factors for recurrent pneumonia, whereas angiotensin converting enzyme-inhibitors usage was associated with a reduction of the risk of RP (aHR 0.22, 0.05–0.91). The detection of *P. aeruginosa* was significantly associated with RP even after adjusting for chronic pulmonary diseases (aHR = 2.37).

**Conclusions:**

Recurrent pneumonia constitutes a considerable proportion of the pneumonia burden in Japan. A past history of pneumonia, chronic pulmonary disease, inhaled corticosteroid and hypnotic/sedative medication usage and detection of *P. aeruginosa* were identified as independent risk factors for recurrent pneumonia and special attention regarding the use of medications in this vulnerable population is needed to reduce the impact of this disease in aging populations.

**Electronic supplementary material:**

The online version of this article (doi:10.1186/s12890-016-0359-1) contains supplementary material, which is available to authorized users.

## Background

Pneumonia is one of the most common infectious diseases and is now the third leading cause of death in Japan [1]. As populations age, the pneumonia burden is forecasted to increase further [2, 3]. In adults, a prior episode of pneumonia is a major risk for subsequent hospital admissions due to pneumonia [4, 5]. Although several studies have investigated recurrent pneumonia (RP), previous investigations have been limited by methods for data analysis. In addition, previous studies report little experience from Asia, including Japan, which has one of the oldest populations in the world [4, 6–9].

In 2004, a prospective cohort study was used to identify several risk factors for community -acquired pneumonia (CAP) [2]. In addition to older age and a past history of pneumonia, this study found that male gender, smoking, increased alcohol intake as well as co-morbid health conditions such as chronic obstructive pulmonary disease (COPD), lung cancer, chronic heart disease, diabetes mellitus, and a history of stroke were associated with CAP [2]. Recently, studies have focused on potentially modifiable medication-associated risk factors including the use of inhaled corticosteroids, acid-suppressing drugs, and angiotensin converting enzyme-inhibitors (ACE-I) [10–12]. However, a limited number of studies have investigated risk factors for RP [6–9]. To reduce the burden of pneumonia, the identification of modifiable risk factors for RP would aid clinicians in the evaluation and management of patients with pneumonia and help guide programs designed to prevent pneumonia among adults in an aging population. To reveal disease burden of total adult pneumonia in Japan, we have previously conducted a multi-center prospective study, called Adult Pneumonia Study Group – Japan (APSG-J) [3]. The current study is a sub-analysis of APSG-J study with the goal of describing the incidence of RP in Japanese adults and identifying the risk factors associated with pneumonia recurrence.

## Methods

### Study design and patient population

In the context of the APSG-J [3], we conducted sub-analysis of data with further collecting data pertaining to RP from a sub-group of patients as follows. The APSG-J study enrolled adult patients aged ≥ 15 years with physician-diagnosed pneumonia, including pneumonia managed as outpatients, at four community hospitals on four major islands in Japan. In the current study targeting RP, a hospital-based cohort was established at Kameda General Hospital, the largest hospital in the APSG-J with 865 beds and 458 clinicians. Kameda General Hospital provides not only primary medical care but also functions as the sole provider of tertiary medical care for residents in Kamogawa city and the surrounding towns in Chiba Prefecture, Central Japan; 31.1% of the referral population were older than 65 years according to the national population census. All residents have good access to hospital care under the universal health coverage. It was assumed that patients registered at the hospital would return if they became ill again.

Patients who visited Kameda General Hospital between February 1, 2012 and January 31, 2013 were followed prospectively until January 31, 2014 to identify cases of RP. A research clinician independently reviewed medical records of patients diagnosed with pneumonia and conducted an independent review of patients’ thoracic diagnostic images and results (i.e., routine chest radiographs and chest computed tomography [CT] scans). For cases in which a diagnostic discrepancy was found between hospital physicians and the research clinician, the final diagnosis of pneumonia was made by an experienced respiratory consultant. In the original multi-center study, the APSG-J research protocol enrolled patients using the following exclusion criteria: hospital-acquired pneumonia (HAP), pneumonia other than acute infectious pneumonia (i.e., interstitial pneumonia, pulmonary tuberculosis, nontuberculous mycobacterial infection, pneumocystis pneumonia, or drowning). To address RP among patients who recovered from the latest pneumonia event, the current study excluded patients who died in the hospital at first enrolment and patients who experienced an episode of pneumonia within 30 days of the last treatment. We assumed our patient population was less influenced by treatment variables for pneumonia at the entry.

The study was conducted in accordance with the Guidelines for Ethical Aspects in Epidemiological Studies (Ministry of Health, Labor and Welfare, 2008) and was approved by the independent research ethics committees of the Institute of Tropical Medicine, Nagasaki University (Nagasaki, Japan) and Kameda General Hospital [3]. Written informed consent was obtained from the majority of the participants or their guardians. The requirement for obtaining written consent from all participants was waived by all independent research ethics committees because of the study’s observational nature without any deviation from the current medical practice.

### Data collection

To identify eligible RP episodes, we selected only the first qualifying event for each patient. Basic demographic, clinical and laboratory data were prospectively collected at first enrolment by hospital physicians and a research clinician. Variables included age, sex, Eastern Cooperative Oncology Group (ECOG) performance status (PS), co-morbid illnesses (e.g., diabetes mellitus, congestive heart failure, ischemic heart disease, chronic liver disease, chronic renal disease, collagen-vascular disease, dementia, malignancy, bronchial asthma, tuberculosis, and chronic obstructive pulmonary disease), medications (e.g., oral corticosteroids, inhaled corticosteroids, immunosuppressants, ACE-Is, biological products, anti-neoplastic drugs, acid-suppressing drugs, antipsychotic drugs, hypnotics and sedatives), and immunization status [2, 13–16]. In addition, information regarding smoking status, alcohol intake, past history of pneumonia, as well as risk factors for aspiration, including episodes of aspiration, dysphagia, consciousness disturbances, neuromuscular diseases, cerebrovascular diseases, tube feeding, and protracted bedridden status [2, 14,﻿ 17]. We also collected information at the first visit to categorize pneumonia episodes into two categories: CAP and healthcare-associated pneumonia (HCAP) according to the definition of the ATS/IDSA guidelines [18, 19]. The severity of pneumonia at presentation was measured using the CURB scoring system [20, 21].

### Microbiological tests

Bacteriological testing of clinical sputum and blood specimens was performed using standard microbiological procedures. *Streptococcus pneumoniae* antigen in the urine was detected using a rapid immunochromatographic assay (BinaxNOW^™^ *Streptococcus pneumoniae*; Alere Inc, Waltham, MA, USA).

### Statistical methods

Rates of recurrent pneumonia (expressed per 100 person-years of observation) were computed from the date of enrolment until the date of the hospital admission for recurrent pneumonia or the end of follow-up (i.e., January 31, 2014), whichever came first. Because Kameda General Hospital services as the sole provider of tertiary care to Kamogawa City residents, we identified the hospital catchment area population based on census data for Kamogawa City (35,766 total residents; according to National Population Census in 2010). Participants who died due to any cause after the enrolment were treated as censored at the date of death. Cox proportional hazards models were applied to quantify the hazard ratios and the 95% confidence intervals (CI) for RP. The multivariate analysis of risk factors associated with RP included all significant variables identified in the univariate analysis considered to be clinically relevant. In detail, we included following factors in multivariate analysis: male, age group, HCAP, past pneumonia history, albumin, performance status, diabetes mellitus, ischemic heart diseases, collagen disease, dementia, lung cancer, chronic pulmonary diseases, aspiration risk factor, oral corticosteroids, inhaled corticosteroids, immunosuppressants, ACE-Is, statins, biological products, anticancer drugs, acid-suppressing drugs, antipsychotic drugs, hypnotics and sedatives, and other drugs. The microbiological test results were compared between patients with and without RP using a chi-square test or Fisher’s exact test. Statistical tests were conducted with STATA version 12.0 (Stata Corp., College Station, TX, USA). All tests were performed in a two-tailed manner, and statistical significance was determined at the 5% level.

## Results

### Patient characteristics

During the study recruitment period, a total of 1,128 patients were considered for enrolment at the study hospital. A total of 287 patients were excluded (231 patients did not meet the inclusion criteria and 56 patients died during the first pneumonia episode) leaving 841 patients eligible for inclusion in the study analysis. The demographic features of patients at first enrolment are summarized in Table 1. The median age was 73 years (range 15–101 years), and a considerable proportion (47%) of patients were ≥ 75 years old. Over half (54%) of the patients were hospitalized, and 20% had HCAP. A quarter of patients had a previous history of pneumonia. A considerable proportion of patients were under nourished with a body mass index (BMI) < 18 kg/m^2^ and/or a low serum albumin level (Alb) of < 3.5 g/dl. The PS was available for 542 patients; 139 (26%) of these patients (16.5% of all patients) did not have fully independent functional status (PS ≥ 2). Approximately one-quarter of pneumonia cases were classified with a CURB score ≥ 2 [20] The majority (74%) of patients had at least one co-morbidity that was potentially associated with pneumonia; the most frequent co-morbidity was chronic obstructive pulmonary disease, followed by diabetes mellitus and malignancy. One-third of the patients were found to have some aspiration risk. Nearly half (48%) of patients were taking medications potentially associated with pneumonia. The most frequently used medications were acid-suppressing drugs, followed by statins, inhaled corticosteroids, oral corticosteroids, and hypnotics.

**Table 1:** Characteristics of study patients and risk factors for recurrent pneumonia; Cox proportional hazard analysis

Characteristic	Subgroup	n (%)	Crude HR	95% CI		*P* > z	Adjusted HR^d^	95% CI		*P* > z
Male		467 (55.5)	1.27	0.9	1.79	0.167	1.08	0.73	1.59	0.696
Age group (years old)	15–49	137 (16.3)	Ref.							
	50–74	308 (36.6)	3.71	1.58	8.69	0.003	2.04	0.84	4.99	0.116
	≥75	396 (47.1)	6.29	2.75	14.4	<0.001	2.6	1.06	6.43	0.038
HCAP		164 (19.5)	2.05	1.41	2.98	<0.001	1.45	0.92	2.31	0.112
Past Pneumonia History^a^		218 (25.9)	2.86	2.04	4	<0.001	1.95	1.35	2.8	<0.001
Albumin (g/dl)	<3.5	330 (39.2)	1.74	1.23	2.45	0.002				
	Unknown	83 (9.9)	0.39	0.16	0.97	0.042				
Body Mass Index	<18	120 (14.3)	1.82	1.19	2.79	0.006	1.41	0.89	2.23	0.14
	Unknown	221 (26.3)	0.77	0.5	1.19	0.24	0.9	0.57	1.41	0.64
Performance Status	≧2	139 (16.5)	1.97	1.29	3.01	0.002	1.08	0.62	1.9	0.787
	Unknown	299 (35.6)	0.9	0.61	1.33	0.594	0.66	0.42	1.03	0.067
CURB	0	292 (34.7)	Ref.							
	1	301 (35.8)	0.99	0.67	1.46	0.942				
	≧2	208 (24.7)	1.07	0.7	1.65	0.752				
	Unknown	40 (4.8)	0.26	0.06	1.08	0.063				
Comorbidities	Diabetes Mellitus	153 (18.2)	1.42	0.95	2.12	0.085	1.39	0.89	2.18	0.149
	Heart Failure	88 (10.5)	0.79	0.43	1.47	0.46				
	Ischemic Heart Diseases	42 (5.0)	2.05	1.13	3.71	0.017	1.18	0.61	2.29	0.616
	Collagen Diseases	66 (7.9)	2.16	1.33	3.5	0.002	1.57	0.69	3.56	0.282
	Dementia	62 (7.4)	1.59	0.91	2.76	0.1	1.29	0.68	2.43	0.431
	Malignancy without Lung Cancer^b^	130 (15.5)	1.02	0.64	1.64	0.929				
	Lung Cancer	21 (2.5)	2.36	1.04	5.35	0.04	2.63	1.09	6.34	0.031
	Bronchial Asthma	82 (9.8)	1	0.57	1.74	0.998				
	Tuberculosis	30 (3.6)	0.57	0.18	1.8	0.343				
	Chronic Pulmonary Diseases	173 (20.6)	2.83	2.01	3.98	<0.001	1.86	1.24	2.78	0.003
	Others	480 (57.1)	1.23	0.87	1.73	0.246				
Aspiration Risk Factor		236 (28.1)	1.66	1.17	2.36	0.004	1.01	0.65	1.58	0.958
Medication	Oral Corticosteroids	76 (9.0)	2.14	1.33	3.44	0.002	1.48	0.74	2.96	0.263
	Inhaled Corticosteroids	86 (10.2)	2.57	1.71	3.88	<0.001	1.78	1.12	2.84	0.015
	Immunosuppressants	27 (3.2)	2	0.98	4.1	0.056	1.3	0.5	3.36	0.591
	ACE-I^c^	37 (4.4)	0.31	0.08	1.25	0.1	0.22	0.05	0.91	0.037
	Statins	114 (13.6)	0.97	0.59	1.6	0.919	0.95	0.57	1.6	0.854
	Biological Products	11 (1.3)	2.44	0.9	6.61	0.078	1.99	0.62	6.37	0.247
	Anticancer Drugs	29 (3.5)	0.39	0.1	1.58	0.186	0.41	0.1	1.7	0.221
	Acid-Suppressing Drugs	235 (27.9)	1.41	0.99	2.02	0.059	0.97	0.64	1.48	0.893
	Antipsychotic Drugs	44 (5.2)	1.85	1.02	3.35	0.041	1.74	0.91	3.35	0.096
	Hypnotics and Sedatives	70 (8.3)	2.81	1.81	4.37	<0.001	2.06	1.28	3.31	0.003
	Others	498 (59.2)	1.49	1.04	2.12	0.029	1.23	0.82	1.85	0.311

*HR* hazard ratio; *CI* confidence interval

^a^14 patients whose past pneumonia history was not available were assumed to not have a past pneumonia history

^b^Malignancy was defined as a history of cancer or active cancer

^c^Angiotensin converting enzyme inhibitor

^d^HRs were adjusted for all other variables

### Incidence of recurrent pneumonia

Over the entire duration of follow-up, a total of 98 deaths were identified among the study patients. Consequently, a total of 1,048 person-years of observations were made with a median follow-up time of 475 (interquartile range, IQR: 380–595) days. During the follow-up period, 137 (16.3%) patients developed RP. The incidence rate of recurrence was 13.1 (95% CI: 11.1–15.5) per 100 person-years. The median time to recurrence was 196 (IQR: 104–339) days, and 82% of episodes occurred within 1 year of presentation. Forty-nine (36%) patients had more than one recurrence. We estimated the incidence rate by limiting the study patients to only residents of Kamogawa City, the site where the study hospital is located. The incidence rate of RP was slightly higher, 14.8 (95% CI, 11.3–19.3) per 100 person-years.

### Characteristics of patients who developed recurrent pneumonia

The clinical presentations at first enrolment were compared between 137 patients who developed RP and 704 patients who did not develop pneumonia. The frequencies of each symptom were similar, and the severity of RP was similar to the severity of the first episode; the proportion of severe pneumonia (CURB ≥ 2) was 24.7% [20]. The duration of treatment was 8 days as median in both groups with RP (3–38 days) and without RP (1–66 days).

### Risk factors for development of recurrent pneumonia

Table 1 summarizes the results of the risk factor analysis. In the univariate analysis, we found that patients with older age, HCAP, a past pneumonia history, underweight status and fully independent functional status were significantly more likely to have experienced RP (*P* < 0.05). When adjusted for gender, age group, past pneumonia history, aspiration risk, co-morbidities and medications, the association with HCAP, low BMI and high PS was no longer present. The severity of pneumonia measured by the CURB score at study enrolment was not associated with RP.

Our univariate analysis showed that several co-morbidities increased the crude risk of RP. Of these, the risk was greatest for patients with pre-existing chronic pulmonary diseases (OR, 2.83; 95% CI: 2.01–3.98) followed by lung cancer, collagen-vascular disease and ischemic heart diseases. However, in the multivariate analysis, the risk of most of these co-morbidities disappeared, except for lung cancer (OR, 2.63; 95% CI, 1.09–6.34) and chronic pulmonary diseases (OR, 1.86; 95% CI, 1.24–2.78) which included both COPD and bronchiectasis. In multivariate analysis, patients receiving inhaled corticosteroids (OR, 1.78; 95% CI, 1.12–2.84), antipsychotic drugs (OR, 1.74; 95% CI: 0.91–3.35) and hypnotics/sedatives (OR, 2.06; 95% CI, 1.28–3.31) were more likely to have experienced RP. Interestingly, patients taking ACE-Is were significantly less likely to develop RP (OR, 0.22; 95% CI, 0.05–0.91). The use of statins was not associated with a reduction of pneumonia recurrence in either the univariate or multivariate analyses.

In univariate analysis, aspiration increased the risk of RP but this association did not persist in the multivariate analysis. We found that age, HCAP, a past pneumonia history and PS were confounding variables for aspiration risk. We also analyzed the lifestyle risk factors of current smoking, alcohol intake, contact with pets, contact with dust, frequent contact with children (≤5 years), frequent contact with the elderly (≥65 years), the number of individuals in the home, familial history of pneumonia and history of travel within 3 months. However, none of these factors exhibited any significant association with RP (data not shown). The immunization history against influenza and pneumococcus was available only in 53.8% of patients; therefore, this factor was not included in the current model.

Kaplan-Meier survival curves depicting the recurrence of pneumonia in relation to the identified risk factors are shown in Additional file 1: Fig. S1. The survival curves did not differ between the age groups of 50–64 and 65–74 years; therefore, in this analysis, these age groups were combined (data not shown).

### Aetiology

Table 2 shows the frequency of bacteria isolated from sputum cultures. A sputum culture was performed in 736 (88%) patients at first enrolment. The most frequently isolated organism was *Haemophilus influenzae*, although when the results of the urine antigen tests were combined, the frequency of *S. pneumoniae* was similar to that of *H. influenzae*. We compared the bacterial isolates at the first enrolment of 130 patients who developed RP with the remaining 606 patients. Although the distribution of isolated bacteria was similar in both groups, the frequency of *Pseudomonas aeruginosa* and gram negative rods was significantly higher in the patients with RP. The detection of *P. aeruginosa* was strongly associated with chronic pulmonary diseases (*P* < 0.001), and the association of *P. aeruginosa* with RP remained significant even after adjusting for chronic pulmonary diseases (Hazard Ratio = 2.37, *P* = 0.001).

**Table 2:** Bacterial pathogens identified by sputum culture and urinary antigen tests

		All		Patients without recurrent pneumonia		Patients with recurrent pneumonia		
		*n* = 736	%	*n* = 606	%	*n* = 130	%	*P*-value
*Streptococcus pneumoniae*		96	13.04	75	12.38	21	16.15	0.246
*Haemophilus influenzae*		119	16.17	101	16.67	18	13.85	0.428
*Moraxella catarrhalis*		60	8.15	46	7.59	14	10.77	0.229
*Staphylococcus aureus*		59	8.02	43	7.1	16	12.31	0.047
Gram-negative bacilli		100	13.59	73	12.05	27	20.77	0.008
	*Pseudomonas aeruginosa*	48	6.52	31	5.12	17	13.08	0.001
	*Klebsiella pneumoniae*	29	3.94	20	3.3	9	6.92	0.054
	*Escherichia coli*	14	1.9	11	1.82	3	2.31	0.709
	*Enterobacter species*	9	1.22	8	1.32	1	0.77	0.604
	Other GNB	12	1.63	10	1.65	2	1.54	0.927
Others		212	28.8	185	30.53	27	20.77	0.026
*S. pneumoniae* (Culture + Urine Antigen)		124	16.78	100	16.42	24	18.46	0.572

Percentages total more than 100% due to multiple culture results

### Survival prognosis of patients with recurrent pneumonia

**Fig. 1:**
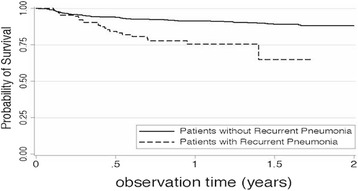
Kaplan-Maier survival curve according to presence of recurrent pneumonia. Patients with recurrent pneumonia were significantly more likely to have fatal outcomes than patients without recurrent pneumonia (Hazard Ratio = 2.81, *P* < 0.001)

Among the 137 patients who developed recurrent pneumonia, 5 patients died during the recurrent episode, and 49 patients experienced another episode of RP. We confirmed a total of 98 deaths at the end of the observation period, of which only 13 patients died during the course of a hospital re-admission for RP. Patients with RP were significantly more likely to have fatal outcomes than patients without RP (Hazard Ratio = 2.81, *P* < 0.001). Figure 1 shows the Kaplan-Meyer survival curves.

## Discussion

### High burden of recurrent pneumonia in Japan

This is the first study demonstrating that RP constitutes a considerable proportion of the pneumonia burden in Japan. One in four pneumonia patients had a past history of pneumonia at study enrolment, and 16% of patients developed RP during the follow-up period of less than two years. This RP rate is higher than the figures of 9.4–12% recently reported in published studies that followed CAP patients for 3–5 years [6, 7]. The higher rate in the present study may have occurred because our study population included more vulnerable individuals, such as elderly subjects, and because we prospectively identified relatively mild pneumonia cases treated in outpatients.

### Risk factors for recurrent pneumonia

The risk factor analysis revealed that in addition to age (≥75 years old), a past pneumonia history, lung cancer, chronic pulmonary diseases, and the administration of inhaled corticosteroids and hypnotics increased the risk of RP. Of these risk factors, a past history of pneumonia was particularly influential because the significant association with HCAP, PS and BMI in the univariate analysis was not present when a past history of pneumonia was integrated into the multivariate model. This strong association with a past history of pneumonia indicates that pneumonia is a reflection of a frail condition and the aggregate of many risk factors of contracting pneumonia.

The risk factors for RP closely overlap but are not identical to risk factors for contracting pneumonia. As Dang, et al. discussed in their paper on RP [7], the selection of the study population on the basis of previous occurrence of pneumonia has potential for introducing bias referred to as index event bias [22]. Due to this type of bias, previously determined risk factors for developing pneumonia, such as aspiration risk [23] and systemic corticosteroid use [24], became less frequently associated with RP. Nevertheless, we believe our risk factor analysis provides a robust multivariate analysis of factors that many clinicians will encounter in their daily practice and provide useful information to clinicians who diagnose pneumonia.

In our analysis, medications represent important, potentially modifiable risk factors for RP. Our study showed that the use of hypnotics was independently associated with RP. Although the association between benzodiazepines and the risk of contracting pneumonia is controversial [25–28], El Solh et al. showed that tranquilizers were a risk factor for recurrent hospitalization for pneumonia among individuals older than 65 years [29], and Dang et al. also reported a tendency of RP in patients using sedative-hypnotics. These findings are consistent with our analysis. Short-acting benzodiazepines or non-benzodiazepine hypnotics are frequently prescribed for elderly patients in Japan [30, 31]. Thus, increased consumption of benzodiazepine sedative-hypnotics may be contributing to the high incidence of RP among elderly Japanese.

Our results are consistent with previous studies [32, 33] showing the use of an inhaled corticosteroids may pose a risk for RP even after adjustment for age and chronic pulmonary diseases. At the same time, we recognize that these drugs consistently decrease COPD exacerbations and improve the quality of life [34, 35] and thus, choices and recommendations regarding the use of inhaled corticosteroids pose a clinical dilemma. These findings suggest that prescribing of inhaled corticosteroids for COPD patients requires special attention when patients have an episode of pneumonia [36].

Our results also indicate that the use of ACE-Is reduce the risk of RP. This finding is consistent with the results of previous papers [7, 29]. ACE-Is have been found to protect against pneumonia, especially among Asians [12, 37]. The protective effects against aspiration by ACE-Is through increasing substance P might become more apparent among a population with dysphagia or silent aspiration, such as our study population. These findings are particularly important for elderly patients suffering from hypertension and other cardiovascular diseases such as congestive heart failure who may be taking ACE-Is on a regular basis.

Eurich, et al. reported that acid-suppressing drugs increased RP during a follow-up period of 5 years [38] but we found no such association after adjustment for age. This discrepant result might be due to that our observation period, a maximum of two years, was too short to demonstrate the effect and that the previous study excluded subjects < 65 years, which were included in our study. Furthermore, another case–control study showed the association of pneumonia was only with proton pump inhibitors but not H2-blockers [39]. Our study could not dissociate the type of acid-suppressing drugs.

### Etiologic agents in recurrent pneumonia

*P. aeruginosa* was the only cultured pathogen that was significantly associated with pneumonia recurrence. Because we defined all culture positive results as *P. aeruginosa* positive cases regardless of patient conditions or bacterial loads, the isolation of *P. aeruginosa* does not necessarily represent infection but could represent colonization. However, in the present study, this association remained significant even after adjustment for all possible risk factors, including chronic pulmonary diseases. Furthermore, the tendency did not change when we re-classified *P. aeruginosa* positive cases, taking into account the quality of sputum and/or the bacterial load and re-analysed the data. This finding suggests the possibility that *P. aeruginosa* plays a role in the pathogenesis of RP. This may suggest that infection/colonization by *P. aeruginosa* play a significant role in the evolution of lung diseases perhaps by interfering the lung microbiome [40]. In addition, patients who are at increased risk for *P. aeruginosa* colonization or infection might also have underlying defects in local immune or mechanical barrier functions, which we did not explore [41]. Gram negative rods were more frequently detected in RP, probably because the risk factors with RP overlap with risk factors of having gram negative rods, such as high age, low performance status with aspiration risk [23, 42, 43] and chronic pulmonary diseases [43]. The corresponding rate of sputum culture based *S. pneumoniae* was 13.0% for all patients and 16.5% for RP group (Table 2), which are low but compatible with our previous report [3]. Furthermore detection rate of pneumococcus would be higher with PCR. These findings of etiology pattern may reflect the nature of pneumonia in an aging population.

### Limitations

Our study has several limitations. First, to estimate the incidence of RP, we assumed that patients registered at the Kameda General Hospital would visit the hospital if they became ill again and seek for treatment because they have good access to hospital-case with same self-pay as the General Practitioner under the universal health coverage; otherwise, they were considered as alive on the census day. However, if frail patients did not seek hospital treatment and died of RP outside Kameda General Hospital, these number were not captured in the outcome analysis. We also excluded patients who died at first enrolment because one of objectives of the study is to clarify the risk factors for RP after appropriate treatment. Therefore, our estimation of the incidence rate and mortality might be underestimated, but the disease burden of RP shown here is still high. Second, this study was analysed using data from a single centre. Third, data were missing from several variables in the current study, despite the baseline APSG-J study was conducted in a prospective manner. This was because these variables were not included in the original APSG-J study design thus it required retrospective data collection through medical chart review. Fourth, unmeasured confounding factors (including demographic characteristics such as socioeconomic status or clinical characteristics and treatment variables such as ICU admission, type of antibiotics) could influence our findings.

## Conclusion

In summary, the present study showed the substantially high disease burden of RP and several independent factors associated with RP, including some medications such as hypnotics, inhaled corticosteroids and ACE-Is, and *P. aeruginosa* infection/colonization. We believe that knowledge of these risk factors might be useful for identifying high risk groups and taking measures to prevent repetitive attacks of pneumonia.

## Additional file

Additional file 1: Figure S1. Kaplan-Meier survival curves for recurrent pneumonia by age group (A), past pneumonia history (B), lung cancer (C), chronic pulmonary diseases (D), inhaled corticosteroid (ICS) (E), hypnotic (F) and angiotensin converting enzyme-inhibitor (ACE-I) use (G). (TIF 12267 kb)
