# Supplementary figures and images for: Recurrent pneumonia among Japanese adults: disease burden and risk factors
Source: BMC Pulm Med. 2017 Jan 11;17:12. doi: 10.1186/s12890-016-0359-1 (PMC5225545; doi:10.1186/s12890-016-0359-1)

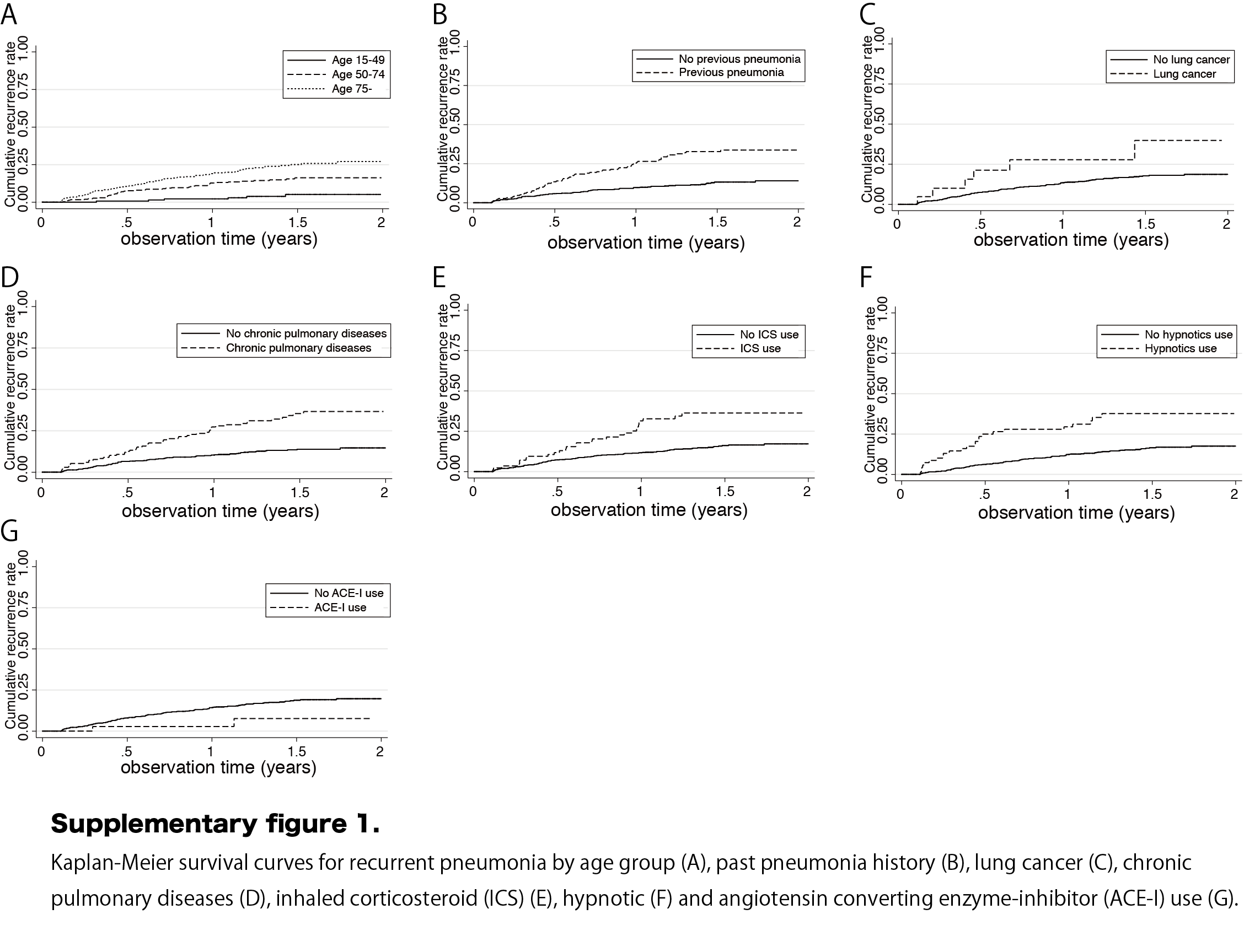

Supplement: Additional file 1: Figure S1. — Kaplan-Meier survival curves for recurrent pneumonia by age group (A), past pneumonia history (B), lung cancer (C), chronic pulmonary diseases (D), inhaled corticosteroid (ICS) (E), hypnotic (F) and angiotensin converting enzyme-inhibitor (ACE-I) use (G). (TIF 12267 kb) [file 12890_2016_359_MOESM1_ESM.tif]
